# Mature Dendritic Cells May Promote High-Avidity Tuning of Vaccine T Cell Responses

**DOI:** 10.3389/fimmu.2020.584680

**Published:** 2020-10-30

**Authors:** Adarsh Kumbhari, Colt A. Egelston, Peter P. Lee, Peter S. Kim

**Affiliations:** ^1^ School of Mathematics and Statistics, University of Sydney, Sydney, NSW, Australia; ^2^ Department of Immuno-Oncology, Beckman Research Institute, City of Hope, Duarte, CA, United States

**Keywords:** T-cell avidity, DC vaccines, cancer vaccines, immature DCs, mathematical model

## Abstract

Therapeutic vaccines can elicit tumor-specific cytotoxic T lymphocytes (CTLs), but durable reductions in tumor burden require vaccines that stimulate high-avidity CTLs. Recent advances in immunotherapy responses have led to renewed interest in vaccine approaches, including dendritic cell vaccine strategies. However, dendritic cell requirements for vaccines that generate potent anti-tumor T-cell responses are unclear. Here we use mathematical modeling to show that, counterintuitively, increasing levels of immature dendritic cells may lead to selective expansion of high-avidity CTLs. This finding is in contrast with traditional dendritic cell vaccine approaches that have sought to harness ex vivo generated mature dendritic cells. We show that the injection of vaccine antigens in the context of increased numbers of immature dendritic cells results in a decreased overall peptide:MHC complex load that favors high-avidity CTL activation and expansion. Overall, our results provide a firm basis for further development of this approach, both alone and in combination with other immunotherapies such as checkpoint blockade.

## Introduction

In principle, the immune system can eliminate cancer cells by the activation and expansion of cancer-specific cytotoxic T lymphocytes (CTLs). Immune checkpoint blockade (ICB) immunotherapies, which release T cells from various negative regulatory pathways, have demonstrated impressive clinical successes and have become standard-of-care for many malignancies (1). However, the response to ICB seems to require the pre-existence of anti-tumor T cells (2). Vaccine approaches to generate tumor-specific T cells offer a potential solution towards generating a sufficient anti-tumor T cell response. Dendritic cell (DC) vaccines in particular, offer a means to activate and expand tumor-specific T cells (3). Here we discuss the impact of DC maturation status on vaccine design strategies.

CTLs detect cancer cells by T cell receptor (TCR) recognition of peptides displayed by a major histocompatibility complex (pMHC) on the surface of target cancer cells. Each TCR-pMHC interaction occurs at a particular strength–*affinity*–with multiple TCR-pMHC interactions occurring for each CTL-target cell interaction. While affinity is a measure of individual TCR-pMHC bonds, *avidity* is an overall measure of the strength of the TCR-pMHC interaction and as such, depends on the amount of pMHC expressed by antigen presenting cells (4). Importantly, T cell avidity determines the likelihood of successful lysis (5).

Therapeutic peptide vaccines aim to capitalize on the cancer-killing ability of CTLs. Initial results of peptide-based vaccines showed the ability to elicit significant numbers of antigen-specific CTLs, but often lacked measurable clinical successes (6–8). Recent progress in vaccine construction and combinatorial strategies with other immunotherapy agents has shown renewed promise for therapeutic peptide vaccines (3). Our work suggests that the dose and modality of peptide vaccines are key considerations for the design of future clinical interventions.

Early studies of cancer-specific CTLs showed that high-avidity TCRs are necessary to effectively lyse cancer cells that express native antigens at low levels (9). Preferentially selecting for high-avidity CTLs, however, is difficult. Regarding vaccines targeting cancer-associated antigens (CAA), thymic education of CTLs may likely have removed high-avidity T cells from the T-cell repertoire *via* negative selection (10). As a result, primarily low-avidity CTLs are left to respond to CAA-targeting vaccines. Beyond CAA, recent therapeutic vaccine efforts have focused on targeting somatic mutation-derived neo-antigens (11, 12). As yet, neo-antigen vaccines have largely focused on peptides sought to elicit high affinity TCR responses but have not yet explored the impact of dosage on T-cell repertoire response to the vaccine (13, 14). For both CAA and neo-antigen targeting vaccines, standard dosages typically involve high antigen loads that may non-discriminately favor the expansion of both high and low avidity CTLs. However, lowering the dosage of peptides for vaccination yields sub-therapeutically relevant levels of CTL (15). Together, this highlights the need for further understanding of antigen dosage and context for efficacious vaccine design.

We previously showed that therapeutic vaccine designs were sensitive to DC-associated parameters (16). Given that DCs, which present antigen on their cell surface along with co-stimulatory molecules, facilitate CTL activation, we hypothesized that modulation of DC and peptide dosing could enhance an anti-cancer immune response. We show that by increasing the number of immature DCs (iDCs), the average DC antigen load is lowered, which in turn selects for the expansion of high-avidity CTLs. This observation suggests traditional DC vaccine approaches that intravenously inject ex vivo matured DCs (mDCs) may need to be reconsidered in favor of an injection of iDCs paired with injection of peptide and adjuvant (3, 17). Our work suggests that combinatorial therapy with vaccine antigens and increased immature DCs, either by ex vivo generation or stimulated *in vivo*, may have efficacy. Thus, our findings suggest an approach that could improve already existing immune-based cancer therapies for increased and more durable clinical responses.

## Material and Methods

We previously developed a mathematical model to study how vaccine-induced avidity selection affects tumor clearance (16). This model was calibrated to ex vivo human data from Chung et al. (18) and then validated against data from (19, 20). Here, we extend this model to show that induction of immature DCs may improve current treatments by eliciting high-avidity CTLs. What follows is a brief description of our previously published model. We primarily use parameter estimates from the literature (see **Table 1** and the references therein) and estimates generated from our prior analysis of ex vivo human data.

**Table 1:** Estimates that are characterized by human data are marked with a superscript H, while estimates based on murine data are marked with a superscript M.

Parameter	Description	Estimate	Reference
*d_p_*	Peptide decay rate ^V^	6.16/day	(21)
*k_P_*	Mature DC uptake rate ^HV^	3 × 10^−2^ (k/μL)^-1^/day	(22)
*k_Pi_*	Immature DC uptake rate ^MV^	6.84 × 10^−2^ (k/μL)^-^/day	(23)
*k_Pϕ_*	Vaccine clearance rate due to tissue macrophages ^MV^	3.1875/day	(24)
*δ_D_*	Immature DC decay rate ^HV^	5 × 10^−2^/day	(25)
*S_D_*	Immature DC supply rate	*δ_D_I *(0)	Steady state
*I*(0)	Immature DC concentration	5.9976 k/μL	(26)
*d_A_*	Adjuvant washout rate ^M^	0.396/day	(27)
*k_S_*	Semi-matured/tolerized DC maturation rate	5 × 10^6^/day	Estimate
*A*_0_	Adjuvant saturation constant	10^4^ ng/mL	Estimate
*d_D_*	Mature DC decay rate ^HV^	0.33/day	(25)
*χ*	Concentration of non-vaccine-associated proteins ^H^	7 × 10^7^ ng/mL	(28)
*k_D_*	Mature DC presentation rate ^MV^	2.4 × 10^5^ pMHCs/day	(29, 30)
*d_m_*	pMHC degradation rate ^MV^	2.9/day	(31)
*N*	(Computational) maximum number of vaccine-associated pMHCs on a maturing DC	700	(16)
*J*	Number of avidity levels	20	(16)
*d_N_N*(0)	Naive CTL supply rate		Steady state
*S_H_*	Naive helper T cell supply rate	*d_NH_N^H^*(0)	Steady state
*S_R_*	Naive nTreg supply rate	*d_NR_N_R_*(0)	Steady state
*d_N_*	Naive CTL egress rate ^M^	1.2/day	(32)
*d_NH_*	Naive helper T cell egress rate ^M^	2.2/day	(32)
*d_NR_*	Naive nTreg turnover rate ^M^	2.2 × 10^-3^/day	(33)
*N*(0)	Initial naive CTL concentration ^M^	7.6 × 10^-3^ k/μL	(34–37)
*N^H^*(0)	Initial naive helper T cell concentration ^M^	0.0571 k/μL	(34, 38)
*N_R_*(0)	Initial naive nTreg concentration ^M^	0.05 × *N^H^*(0)	(39)
*R*_LH_	Ratio of low-high avidity naive CTLs	100	Assumption
*n_R_*	Number of nTreg divisions ^HV^	6	(40)
*k*_DC_	Naive CTL-DC interaction rate ^M^	0.4 (k/μL)^-1^/day	(34)
*τ_m_*	DC migration time ^M^	0.75 days	(34)
*V*_tissue_	Volume of tissue site	1000 μL	(16)
*V*_LN_	Volume of lymph node ^M^	4.2 μL	(34)
*n_H_*	Number of helper T cell divisions ^HV^	10	(41)
*τ_a_*	T cell division time ^M^	1 day	(38, 42)
*d_H_*	Effector helper T cell decay rate ^H^	0.008/day	(43)
*n_T_*	Number of CTL divisions ^M^	15	(42, 44–47)
*d_T_*	Effector CTL decay rate ^H^	0.009/day	(43)
*φ_0_*	Antigen saturation constant	5 × 10^3^ ng/mL	(16)
*r*_1_	Secretion rate of growth signal by CTLs	0.1/day	(16)
*r*_2_	Secretion rate of growth signal by helper T cells	1/day	(16)
*d_G_*	Growth factor decay rate ^H^	144.4/day	(48)
*k_G_*	T cell-growth factor interaction rate	0.1 (k/μL)^-1^/day	(16)
*k_R_*	iTreg differentiation rate	0.2/day	(16)
*d_R_*	iTreg decay rate ^H^	0.083/day	(49, 50)
*d_RN_*	Effector nTreg decay rate ^H^	0.063/day	(50)
*μ*	CTL-Treg interaction rate ^H^	5 (k/μL)^-1^/day	(16)
*K*	(Computational) maximum number of cognate pMHCs expressed on cancer cell	295	(16)
*γ*	Growth rate of melanomas ^H^	0.0185/day	(51–53)
*κ*	Carrying capacity of melanomas ^M^	736 k/μL	(54)
*α*	pMHC regeneration rate ^MV^	8.4/day	(55, 56)
*k_T_*	Tumor-CTL interaction rate ^HV^	16.1 (k/μL)^-1^/day	Estimate
*p_T_*	Probability of trogocytosis ^HV^	0.7	Estimate
*ω*_1_	Lysis likelihood for lowest avidity (*j* = 1) CTL ^HV^	0.28	Estimate
*ω_j_*	Lysis likelihood for highest avidity (*j* = *J*) CTL ^HV^	0.96	Estimate
*C*_init_	Initial cancer concentration	0.05 k/μL	(16, 18)

Additionally, estimates that are based on cell culture data are marked with a superscript V. Finally, the unit ‘k’ denotes 10^3^ cells.

### Basic Model

**Figure 1:**
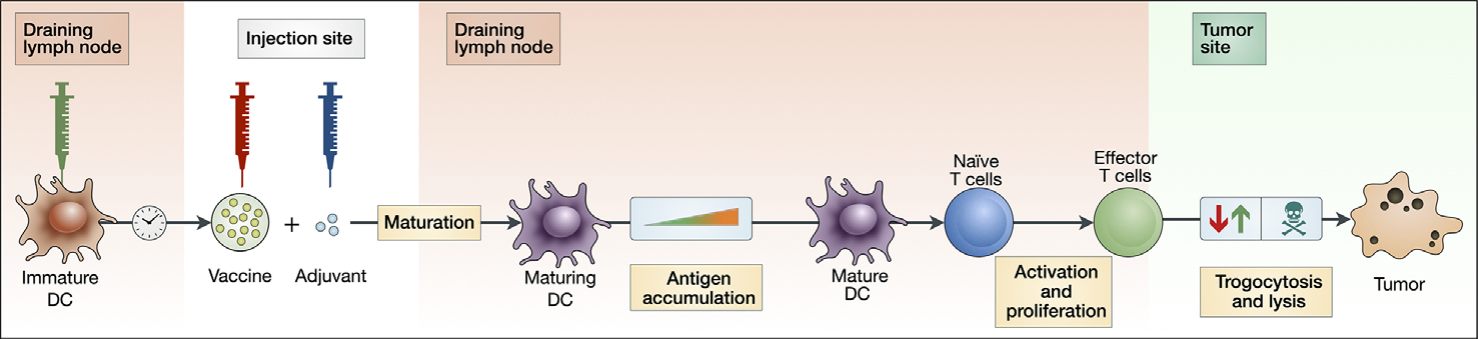
Block diagram depicting key aspects of our theoretical vaccination model. An injection of peptide vaccine is given intramuscularly with adjuvant. Immature DCs are injected intranodally, prompting an accumulation of antigen by maturing DCs. These maturing DCs then migrate and activate naive T cells in the lymph node, which then proliferate into effector T cells. Effector T cells can both strip peptides off the surface of cancer cells *via* trogocytosis and kill cancer cells.

The model consists of three major components: the activation and maturation of DCs (Eqs 1–8); the activation and proliferation of T cells (Eqs 9–16); and the lysis and trogocytosis-mediated MHC stripping of cancer cells by effector CTLs (Eqs 23–25). **Figure 1** depicts a schematic of these interactions.

#### Dendritic Cells

To model the activation and maturation of DCs at the injection site (the volume of which is V_tissue_), we consider several populations: *P*, the concentration of vaccine peptides; *A*, the concentration of vaccine adjuvant; *I*, the concentration of immature DCs; *S*, the concentration of semi-mature or “tolerizing” DCs; and *M_j_*, the concentration of maturing DCs presenting *j* vaccine-associated pMHCs, where *j* can vary between zero and *N*. In modelling the interactions between these populations, we assume that immature DCs become semi-mature in the presence of peptide antigen, various danger signals, and tissue-derived immunogenic signals (57, 58). Once in this semi-mature state, we assume DC maturation occurs as a result of vaccine adjuvant. DC maturation signals may in turn affect T-cell priming and activation (19). As a simplifying assumption, we assume that the strategy to optimize DC maturation is successful. That is, we do not model the pharmacodynamics of the vaccine adjuvant. Next, we model the interactions between these populations with an ODE system:

Change in vaccine peptide concentration:

(1) $$\frac{dP}{dt}=\underset{\begin{array}{c} Vaccine\\ injection \end{array}}{\underset{︸}{u(t)}}-\underset{Vaccine\;turnover}{\underset{︸}{d_{P}P}}-\underset{Uptake\;by\;iDCs}{\underset{︸}{k_{Pi}PI}}-\underset{Uptake\;by\;mDCs}{\underset{︸}{k_{P}P\sum_{j=0}^{N}M_{j}}}-\underset{\begin{array}{c} Turnover\;due\;to\\ \;macrophage\;uptake \end{array}}{\underset{︸}{k_{P\phi }P,}}$$

Change in adjuvant concentration:

(2) $$\;\frac{dA}{dt}=\underset{Adjuvant\;injection}{\underset{︸}{a(t)}}-\underset{Uptake\;by\;iDCs}{\underset{︸}{k_{Pi}AI}}-\underset{Adjuvant\;washout}{\underset{︸}{d_{A}A}}-\underset{\begin{array}{c} Turnover\;due\;to\\ \;macrophage\;uptake \end{array}}{\underset{︸}{k_{P\phi }A,}}$$

Change in immature DC concentration:

(3) $$\frac{dI}{dt}=\underset{iDC\;\text{supply}}{\underset{︸}{s_{D}}}-\underset{iDC\;turnover}{\underset{︸}{\delta_{D}I}}-\underset{iDC\;activation}{\underset{︸}{k_{D}\frac{P}{\chi +P}I,}}$$

Change in semi-mature/tolerized DC concentration:

(4) $$\;\frac{dS}{dt}=\underset{iDC\;activation}{\underset{︸}{k_{D}\frac{P}{\chi +P}I}}-\underset{Turnover}{\underset{︸}{d_{D}S}}-\underset{\begin{array}{c} Maturation\;due\;to\\ adjuvant \end{array}}{\underset{︸}{k_{S}\frac{A}{A+A_{0}}S,}}$$

Change in mature DC concentration:

(5) $$\frac{dM_{0}}{dt}=-k_{D}\frac{P}{\chi +P}M_{0}+d_{m}M_{1}-d_{D}M_{0},$$

(6) $$\frac{dM_{1}}{dt}=\underset{\begin{array}{c} Maturation\;due\;to\\ adjuvant \end{array}}{\underset{︸}{k_{S}\frac{A}{A+A_{0}}S}}+\underset{pMHC\;presentation}{\underset{︸}{k_{D}\frac{P}{\chi +P}\left(M_{0}-M_{1}\right)}}+\underset{pMHC\;turnover}{\underset{︸}{d_{m}\left(2M_{2}-M_{1}\right)}}-\underset{mDC\;turnover}{\underset{︸}{d_{D}M_{1},}}$$

(7) $$\frac{dM_{j}}{dt}=\underset{\text{pMHC presentation}}{\underset{︸}{k_{D}\frac{P}{\chi +P}\left(M_{j-1}-M_{j}\right)}}+\underset{\text{pMHC turnover}}{\underset{︸}{d_{m}\left(\left(j+1\right)M_{j+1}-jM_{j}\right)}}-\underset{\text{mDC turnover}}{\underset{︸}{d_{D}M_{j},}}\;\text{for }j=2,\ldots ,N-1,$$

(8) $$\frac{dM_{N}}{dt}=k_{D}\frac{P}{\chi +P}M_{N-1}-Nd_{m}M_{N}-d_{D}M_{N}.$$

In Equation 1, vaccine peptides are injected intramuscularly at rate *u*(t), decay at rate *d_p_*, taken up by immature DCs at rate *k_Pi_*, taken up by mature DCs at rate *k_p_*, and are competitively diminished due to consumption by tissue macrophages at rate *k_P_*_ϕ_ (note that we do not model these macrophages in our study). Here, we assume that iDCs have a greater antigen uptake rate than mDCs (59, 60). In Equation 2, vaccine adjuvants are injected intramuscularly at rate a(t), taken up by immature DCs at rate *k_Pi_*, washed out at rate *d_A_*, and are lost due to consumption by splenic macrophages at rate *k_P_*_ϕ_. In Equation 3, immature DCs are supplied at rate *s_D_*, decay at rate $\delta_{D}$ and become semi-mature and acquire vaccine peptides at rate $k_{D}\frac{P}{\chi +P}.$ Here, *k_D_* is the rate of peptide presentation, *χ* is the concentration of non-vaccine peptides, and $\frac{P}{\chi +P}$ is the proportion of peptides presented that are vaccine specific.

In Equation 4, we assume that semi-mature DCs, *S*, turnover at a rate comparable to mature DCs *d_D_*, and mature due to adjuvant at rate $k_{S}\frac{A}{A+A_{0}}.$ Here, $k_{S}$ is the maturation rate due to adjuvant and $A_{0}$ is a adjuvant-saturation constant that ensures that for large adjuvant doses, the DC maturation tapers off. In the absence of adjuvant, however, these semi-mature DCs are unlikely to produce a functional T cell response (61). Thus, for the purposes of this study we do not track T cells that become tolerized as a result of these semi-mature/tolerized DCs.

In Eqs 5 and 6, newly matured DCs initially enter the mature DC population presenting one vaccine peptide with subsequent peptides presented at rate $k_{D}\frac{P}{\chi +P}$ as described above. Additionally, surface peptides degrade at rate *d_m_*, which is proportional to the number of presented peptides, *j*. Finally, mature DCs decay at rate *d_D_*. Here, we assume that mature DCs decay faster than iDCs (62).

#### T Cells

To model the activation and proliferation of T cells both at the lymph node (the volume of which is *V*_LN_) and at the tumor site, we first model avidity as a spectrum that varies from *j*=1 to *j*=*J*, corresponding to the lowest and highest avidity states respectively. We then consider several populations: *N_j_*, the concentration of naive CTLs of avidity *j*; $N_{j}^{H}$, the concentration of naive helper T cells of avidity *j*; *N_R_*, the concentration of naive natural regulatory T cells; *T_j_*, the concentration of effector CTLs of avidity *j*; *H_j_*, the concentration of effector helper T cells of avidity *j*; *R*, the concentration of induced regulatory T cells; *R_N_*, the concentration of effector natural regulatory T cells; and *G*, the concentration of positive growth factors. The interactions between these populations are then modelled with an ODE system:

Change in naive helper T cell concentration:

(9) $$\;\;\frac{dN_{j}}{dt}=\underset{\text{Supply}}{\underset{︸}{\rho_{j}s_{T}}}-\underset{\text{Turnover}}{\underset{︸}{d_{N}N_{j}}}-\underset{\text{Activation}}{\underset{︸}{\frac{V_{\text{tissue}}}{V_{\text{LN}}}e^{-d_{D}{\text{τ}}_{m}}\sum_{k=1}^{N}\left(k_{\text{DC}}p_{j,k}\right)N_{j}M_{k}\left(t-{\text{τ}}_{m}\right)}},$$

Change in naive killer T cell concentration:

(10) $$\frac{dN_{j}^{H}}{dt}=\underset{\text{Supply}}{\underset{︸}{{\text{ρ}}_{j}s_{H}}}-\underset{\text{Turnover}}{\underset{︸}{d_{NH}N_{j}^{H}}}-\underset{\text{Activation}}{\underset{︸}{\frac{V_{\text{tissue}}}{V_{\text{LN}}}e^{-d_{D}{\text{τ}}_{m}}\sum_{k=1}^{N}\left(k_{\text{DC}}p_{j,k}\right)N_{j}^{H}M_{k}\left(t-{\text{τ}}_{m}\right)}},$$

Change in naive natural regulatory T cell concentration:

(11) $$\;\frac{dN_{R}}{dt}=\underset{\text{Supply}}{\underset{︸}{s_{R}}}-\underset{\text{Turnover}}{\underset{︸}{d_{NR}N_{R}}}-\underset{\text{Activation}}{\underset{︸}{\frac{V_{\text{tissue}}}{V_{\text{LN}}}e^{-d_{D}{\text{τ}}_{m}}\sum_{k=1}^{N}\;\sum_{j=1}^{J}\left(k_{\text{DC}}p_{j,k}\right)N_{R}M_{k}\left(t-{\text{τ}}_{m}\right)}},$$

Change in effector killer T cell concentration:

(12) $$\frac{dT_{j}}{dt}=\begin{array}{l} \overset{T\;cell\;induction}{\overset{︷}{e^{-d_{N}{\text{τ}}_{a}}\varphi (P)2^{n_{T}}e^{-d_{D}{\text{τ}}_{m}}\sum_{k=1}^{N}\left(k_{DC}p_{j,k})\;N_{j}(t-{\text{τ}}_{a})M_{k}(t-{\text{τ}}_{m}-{\text{τ}}_{a}\right)}}-\overset{Turnover}{\overset{︷}{d_{T}T_{j}}}-\overset{iTreg\;suppression}{\overset{︷}{\mu RT_{j}}}-\;\underset{nTreg\;suppression}{\underset{︸}{\mu R_{N}T_{j}}}+\underset{Growth\;due\;to\;growth\;factors}{\underset{︸}{k_{G}GT_{j},}} \end{array}$$

Change in effector helper T cell concentration:

(13) $$\frac{dH_{j}}{dt}=\begin{array}{l} \overset{Tcellinduction}{\overset{︷}{e^{-d_{NH}{\text{τ}}_{a}}2^{n_{H}}e^{-d_{D}{\text{τ}}_{m}}\sum_{k=1}^{N}\left(k_{DC}p_{j,k})\;N_{j}^{H}(t-{\text{τ}}_{a})M_{k}(t-{\text{τ}}_{m}-{\text{τ}}_{a}\right)}}-\overset{\begin{array}{l} Differentiation\\ intoiTregs \end{array}}{\overset{︷}{k_{R}H_{j}}}-\overset{iTreg\;supression}{\overset{︷}{\mu RH_{j}}}-\;\underset{nTreg\;supression}{\underset{︸}{\mu R_{N}H_{j}}}+\underset{Turnover}{\underset{︸}{d_{H}H_{j}}}\underset{Growthduetogrowthfactors}{\underset{︸}{k_{G}GH_{j},}} \end{array}$$

Change in natural regulatory T cell concentration:

(14) $$\;\frac{dR_{N}}{dt}=\underset{\text{nTreg induction}}{\underset{︸}{e^{-d_{NR}\tau_{a}}2^{n_{R}}e^{-d_{D}\tau_{m}}\sum_{k=1}^{N}\;\sum_{j=1}^{J}\left(k_{\text{DC}}p_{j,k}\right)N_{R}\left(t-{\text{τ}}_{a}\right)M_{k}\left(t-\tau_{m}-\tau_{a}\right)}}-\underset{\text{Turnover}}{\underset{︸}{d_{RN}R_{N}}},$$

Change in induced regulatory T cell concentration:

(15) $$\;\;\;\;\frac{dR}{dt}=\underset{\begin{array}{c} \text{Differentiated}\\ \text{helper T cells} \end{array}}{\underset{︸}{k_{R}\sum_{j=1}^{J}H_{j}}}-\underset{\text{Turnover}}{\underset{︸}{d_{R}R}},$$

Change in concentration of positive growth factors:

(16) $$\;\;\;\;\frac{dG}{dt}=\underset{\begin{array}{c} \text{Secretion}\\ \text{by CTLs} \end{array}}{\underset{︸}{r_{1}\sum_{j=1}^{J}T_{j}}}+\underset{\begin{array}{c} \text{Secretion by}\\ \text{helper T cells} \end{array}}{\underset{︸}{r_{2}\sum_{j=1}^{J}H_{j}}}-\underset{\begin{array}{c} \text{Consumption}\\ \text{by T cells} \end{array}}{\underset{︸}{k_{G}G\sum_{j=1}^{J}\left(T_{j}+H_{j}\right)}}-\underset{\text{Turnover}}{\underset{︸}{d_{G}G}}.$$

In Equation 9, naive CTLs in the lymph node of avidity *j* are supplied at rate *ρ_j_s_T_*, where *ρ_j_* is the proportion supplied that have avidity *j*. These naive CTLs also exit the lymph node at rate *d_N_*. The rate at which naive CTLs are activated by mature DCs that have migrated into the lymph node is

(17) $$\begin{array}{c} \frac{V_{\text{tissue}}}{V_{\text{LN}}}e^{-d_{D}{\text{τ}}_{m}}\sum_{k=1}^{N}\left(k_{\text{DC}}p_{j,k}\right)N_{j}M_{k}\left(t-{\text{τ}}_{m}\right).\; \end{array}$$

Migration is modelled with a fixed delay of *τ_m_*, with $e^{-d_{D}\tau_{m}}$ being the proportion that survives migration. For intranodal injections, the value of *τ_m_* is set to zero. The kinetic interaction rate between naive CTLs of avidity *j* and mature DCs presenting *k* vaccine-peptides is *k*_DC_ with *p_j,k_* being the probability of an interaction leading to successful activation. This means the *net* kinetic rate, *k*_DC_ *p_j,k_*, depends on both T cell avidity, *j*, and the number of pMHCs presented on a DC, *k*. Finally, the leading term $\frac{V_{\text{tissue}}}{V_{\text{LN}}}$ accounts for the volume change between the injection site and the lymph node. However, for intranodal injections, this ratio is set to one as there will be no change in volume. In Equation 10, which is similar to Equation 9, naive helper T cells of avidity *j* are supplied at rate *ρ_j_s_H_*, decay at rate *d_NH_*, and are activated at the net rate of

(18) $$\begin{array}{c} \frac{V_{\text{tissue}}}{V_{\text{LN}}}e^{-d_{D}{\text{τ}}_{m}}\sum_{k=1}^{N}\left(k_{\text{DC}}p_{j,k}\right)N_{j}^{H}M_{k}\left(t-{\text{τ}}_{m}\right). \end{array}$$

In Equation 11, which is similar to Eqs 9 and 10, naive natural regulatory T cells (nTregs) are supplied at rate *s_R_*, decay at rate *d_NR_*, and are activated at the net rate of

(19) $$\begin{array}{c} \frac{V_{\text{tissue}}}{V_{\text{LN}}}e^{-d_{D}{\text{τ}}_{m}}\sum_{k=1}^{N}\;\sum_{j=1}^{J}\left(k_{\text{DC}}p_{j,k}\right)N_{R}M_{k}\left(t-{\text{τ}}_{m}\right). \end{array}$$

As we do not account nTregs of different avidities, we sum over the variable *j*.

Equations 12–16 describe interactions within the tumor site. In Equation 12, naive CTLs undergo *n_T_* divisions. The division program is modelled with a fixed delay of *τ_a_*, with $e^{d_{N}\tau_{a}}$ being the proportion that effectively activate and traffic to the tumor site. As a consequence, not all T cells that exit the lymph node arrive as effector T cells at the tumor site. These assumptions equate to a net supply rate of

(20) $$\begin{array}{c} e^{-d_{N}\tau_{a}}2^{n_{T}}k_{\text{DC}}e^{-d_{D}\tau_{m}}\sum_{k=1}^{N}p_{j,k}N_{j}\left(t-\tau_{a}\right)M_{k}\left(t-\tau_{m}-\tau_{a}\right). \end{array}$$

To account for T-cell hyporesponsiveness, we multiply Equation 20 by $\varphi (P)=\frac{\varphi_{0}}{\varphi_{0}+\int_{0}^{t}P\;(s)\;ds}.$ This ensures that antigen accumulation results in diminished effector CTL expansion. We also assume effector CTLs: decay at rate *d_T_*; expand due to interactions with positive growth factors at rate *k_G_*; and are suppressed by interactions with induced regulatory T cells at rate *μ*. Given that induced regulatory T cells (iTregs) and effector nTregs have similar suppression rates (63, 64), we assume that nTregs suppress effector CTLs at an identical rate of *μ*.

In Equation 13, naive helper T cells undergo *n_H_* divisions. Following a similar argument to that in Equation 12, the net supply rate of effector helper T cells is

(21) $$\begin{array}{c} e^{-d_{NH}\tau_{a}}2^{n_{H}}k_{\text{DC}}e^{-d_{D}\tau_{m}}\sum_{k=1}^{N}p_{j,k}N_{j}\left(t-\tau_{a}\right)M_{k}\left(t-\tau_{m}-\tau_{a}\right). \end{array}$$

These effector helper T cells differentiate into induced regulatory T cells at rate *k_R_*; are suppressed by both iTregs and nTregs at rate *μ*; decay at rate *d_H_*; and expand due to interactions with positive growth factors at rate *k_G_*.

In Equation 14, following a similar argument to that in Equation 12, effector nTregs enter the system at rate

(22) $$\begin{array}{c} e^{-d_{NR}\tau_{a}}2^{n_{R}}e^{-d_{D}\tau_{m}}\sum_{k=1}^{N}\;\sum_{j=1}^{J}(k_{DC}p_{j,k})N_{R}\left(t-\tau_{a}\right)M_{k}\left(t-\tau_{m}-\tau_{a}\right), \end{array}$$

and decay at rate *d_RN_*. In Equation 15, iTregs enter the system as differentiated effector helper T cells and decay at rate *d_R_*. Finally, in Equation 16, effector CTLs and helper T cells secrete growth factors such as IL‑2 at rates *r*_1_ and *r*_2_. These growth factors are assumed to decay at rate *d_G_*.

#### Cancer Cells

To model the lysis of cancer cells and trogocytosis of cancer cell MHC by effector CTLs, we consider a population of cancer cells presenting *k* vaccine-associated peptides, *C_k_*, where *k* varies from zero to *K*. The interactions between these cancer cells and effector CTLs are modelled with an ODE system:

(23) $$\frac{dC_{0}}{dt}=\gamma (1-C_{total}/\kappa )(C_{0}+C_{1})-\alpha C_{0}+k_{T}\left(\sum_{j=1}^{N}T_{j}\right)\left(\sum_{m=1}^{K}C_{m}q_{m,m}\right),$$

For *k* = 1, ···, *K* – 1,

(24) $$\begin{array}{l} \overset{\text{Growth}}{\overset{︷}{\frac{dC_{0}}{dt}=\gamma (1-C_{total}/\kappa )(-C_{k}+2C_{2k}+C_{2k-1}+C_{2k+1})}}+\overset{pMHC\;regeneration}{\overset{︷}{\alpha (C_{k-1}-C_{k})}}\\ \;+\underset{Trogocytosis}{\underset{︸}{k_{T}\left(\sum_{j=1}^{N}T_{j}\right)\left(\left(\sum_{m=k+1}^{K}C_{m}q_{m-k,m}\right)-C_{k}(1-q_{0,k})\right)}}-\underset{Lysis}{\underset{︸}{k_{T}\sum_{j=1}^{N}\lambda_{j,k}T_{j}C_{k},}} \end{array}$$

(25) $$\frac{dC_{k}}{dt}=\gamma C_{k}(1-C_{total}/\kappa )+\alpha C_{K-1}-k_{T}\left(\sum_{j=1}^{N}T_{j}\right)C_{k}(1-q_{0,k})-k_{T}\sum_{j=1}^{N}\lambda_{j,k}T_{j}C_{k}.$$

In Eqs 23–25, the total cancer population, $C_{total}=\Sigma_{k=0}^{K}C_{j,}$ grows logistically at rate *γ* and with carrying capacity κ. As a simplifying assumption, we assume that the number of surface peptides is halved after mitosis, resulting in a net compartmental growth rate of

(26) $$\gamma \left(1-\frac{C_{total}}{\kappa }\right)(-C_{k}+C_{2k}+C_{2k-1}+C_{2k+1}),$$

for the population of cancer cells presenting *k* peptides, *C_k_*. We also assume that surface peptides are regenerated at rate *α*. To model trogocytosis-mediated MHC stripping, we assume that CTLs and cancer cells presenting *k* peptides interact at rate *k_T_* and additionally assume the number of peptides stripped during this interaction is binomially distributed with probability *p_T_*. For brevity we let $q_{m,n}=\left(\begin{array}{c} n\\ m \end{array}\right)p_{T}^{m}{\left(1-p_{T}\right)}^{n-m}$ denote the probability that a CTL will trogocytose *m* MHC:peptides off a cancer cell presenting *n* surface peptides. This allows us to describe the trogocytosis rate as

(27) $$k_{T}(\Sigma_{j=1}^{N}T_{j})\left(\left(\Sigma_{m=k+1}^{K}C_{m}q_{m-k,m})-C_{k}(1-q_{0,k}\right)\right).$$

Finally, to model lysis, we let *λ_j,k_* denote the lysis probability between a cancer cell presenting *k* peptides and an effector CTL of avidity *j* and assume that these interactions occur at rate *k_T_*. This implies the net kinetic interaction rate depends on both T cell avidity and the amount of pMHC presented by a cancer cell. To model the lysis probability, we assume that the probability of lysis increases with cognate pMHCs but is also modulated by CTL avidity. This can be modelled by assuming a probability function of the form

$$1-e^{-r_{j}k},$$

where *r_j_* is an avidity-dependent rate parameter chosen so that the lysis probability at maximal levels of cognate pMHC expression, i.e., *λ_j,k_* varies linearly from *ω*_1_ for the lowest avidity CTL to *ω_J_* for the highest avidity CTL.

### Functional Forms

#### Peptide Vaccine Injection Rate

Here, we assume that the vaccine is injected systemically at a fixed dose, *u*_0_, and at a regular interval of ζ, which corresponds to the functional form

$$u(t)=u_{0}\sum_{a=0}^{\infty }\delta (t-\zeta a).$$

#### Vaccine Adjuvant Injection Rate

We assume that the vaccine adjuvant is injected at a clinically-relevant fixed dose of 5×10^5^ ng/mL (19), and at a regular interval of *η*, corresponding to the functional form

$$a(t)=(5\times 10^{5}\text{ng}/\text{ml})\times \sum_{n=0}^{\infty }\delta (t-n\eta ).$$

#### Peptide Uptake Rates

We previously used ex vivo human data from (22) to estimate a mature DC uptake rate, *k_P_*, of 3 × 10^−2^ (k/μL)^-1^/day. It is generally understood that immature DCs, relative to mature DCs, have a greater uptake rate (59, 60). To estimate the uptake rate by iDCs, we use *in vitro* murine data from (23), who note that antigen internalization (as quantified by staining for the antibody YAe) in iDCs is 2.28 times greater than in mDCs. Thus, we assume that the uptake by iDCs, *k_Pi_*, is 2.28 × *k_P_* = 6.84 × 10^−2^ (k/μL)^‑1^/day. To account for vaccine clearance by splenic macrophage, we first note the steady-state concentration of non-activated macrophages in mice is estimated to be 1.25 × 10^-1^ k/μL (24). In (24), the authors also estimate the rate of phagocytosis by non-activated macrophages to be 25.2 (k/μL)^‑1^/day. Together, these correspond to a splenic macrophage associated vaccine clearance rate, *k_Pϕ_*, of 25.2 (k/μL)^‑1^/day × 0.125 k/μL = 3.1875/day.

#### Activation Probability

The probability of a mature DC presenting *k* vaccine-associated pMHCs activating a naive T cell of avidity *j*, *p_j,k_*, is modelled with a switch:

$$p_{j,k}=\{\begin{array}{ll} 1, & \text{if}\left|\frac{j-1}{J-1}-\left(1-\frac{k-1}{N_{c}-1}\right)\right|\leq v\;and\;k<N_{c}\\ 1, & \text{if }j=1\;and\;k\geq N_{c}\\ 0, & otherwise. \end{array}$$

Here, 1/(*N_c_* – 1) and 1/(*J* – 1) map *j* and *k* from their respective domains to [0,1]. The dimensionless parameter *v* = 0.05 determines how sensitive our switching function is to pMHC expression. This characterization ensures that and high pMHC levels on DCs stimulate both high- and low-avidity CTLs (20, 65–69) and by contrast, low pMHC expression stimulates mostly high-avidity CTLs (10, 70–72). To reflect this, we assumed that beyond a critical number of pMHCs, *N_c_*, only low-avidity CTLs were stimulated. We set *N_c_* =*N* / 2 = 350, implying that DCs must have a surface antigen density *below* 50% to stimulate high-avidity CTLs.

#### Initial Conditions

We assume that the vaccine is first administered at *t* = 0, i.e., *P*(0) = *u_0_*, where *u_0_* is the vaccine dose. Our model assumes a large number of immature DCs preexist at the injection site. In (73), the total DC population at steady-state conditions in the dermis is estimated to be approximately 23.4 k/μL. Around 92.74% of this population is expected to immature (74), equating to an initial iDC concentration at the injection site of 21.7 k/μL. Similarly, 7.26% of the total DC population is expected to be mature (74), equating to a total mDC concentration of 1.7 k/μL. For intravenous injections, we use a total DC concentration of 25 DCs/μL (75), and assume that 90% of this population is immature, equating to an iDC concentration of 22.5 DCs/μL and total mDC concentration of 2.5DCs/μL. Finally, for intranodal injections, we use a pre-existing LN iDC count of 25,190 cells, and an mDC count of 32,920 cells (26), which for a control volume of *V*_LN_, equates to an iDC concentration of 5.9976 k/μL, and a total mDC concentration of 7.8381 k/μL. Moreover, we assume that within this mature DC population, pMHCs are normally distributed with mean *μ* = 100 and variance *σ* ^2^ = 25 (76). As a simplifying assumption, we assume the initial concentration of semi-mature/tolerizing DCs is zero.

To model the scarcity of high-avidity naive T cells, we assume that their availability decreases exponentially. Specifically, we assume *N_j_*(0) = *ρ_j_N*(0) and $N_{j}^{H}(0)=\rho_{j}N^{H}(0),$ where $\rho_{j}=ae^{-bj}.$ Here, the model parameters *a* and *b* are chosen so that $\Sigma_{j=1}^{J}\rho_{j}=1$ and ρ_1_/ρ*_J_*, i.e., the ratio low-avidity to high-avidity T cells, equates to the model parameter *R*_LH_. In our simulations, we set *R*_LH_ to 100, which means that for one high-avidity T cell there are 100 low-avidity T cells. Moreover, naive natural regulatory T cells, *N_R_*, make up roughly 5% of the naive helper T cell population (39), thus, we set $N_{R}(0)=0.05\times N^{H}(0).$

Prior to vaccination, tumor-specific effector T cells exist, albeit at low concentrations (approximately 0.12% of the total CD8+ count) (77). Assuming a total CD8+ count of 600 cells/μL (78), this equates to an initial tumor-specific effector CTL concentration of 0.72 cells/μL. To estimate the initial tumor-specific effector helper T cell concentration, we assume a comparable percentage (i.e., 0.12%) also exists before vaccination. Using a circulating helper T cell concentration of 10^3^ cells/μL (79), this corresponds to an initial tumor-specific effector helper T cell concentration of 1.2 cells/μL. Moreover, approximately 1.5% of this helper T cell pool expresses the natural regulatory T cell phenotype (39), which equates to an initial effector natural regulatory T cell concentration of *R_N_* (0) = 1.8 × 10^-2^ cells/μL. As a simplification, we assume that initially there are no induced regulatory T cells, i.e., *R* (0) = 0, and that the concentration of growth factor is zero, i.e., *G* (0) = 0. Finally, to account for the scarcity of high-avidity T cells, we multiply the concentrations of effector CTLs and effector helper T cells by *ρ_j_* (defined in the above paragraph). Mathematically, *T_j_* (0) = *ρ_j_* × 0.72 cells/μL and *H_j_* (0) = *ρ_j_* × 1.2 cells/μL. In other words, initially, for every high-avidity tumor-specific effector T cell, there are 100 low-avidity tumor-specific effector T cells.

Finally, we assume that the total cancer cell concentration is *C*_init_, with cognate pMHC being normally distributed with mean *μ* = 148 and variance *σ* ^2^ = 49. Mathematically, if $f_{k}=\frac{1}{\sigma \sqrt{2\pi }}\exp\left(-\frac{{\left(k-\mu \right)}^{2}}{2\sigma^{2}}\right),$ then $C_{k}(0)=C_{init}\times \frac{f_{k}}{\Sigma_{k=1}^{K}f_{k}}.$

### Sensitivity Analysis

To understand how DC maturation status affects parameter sensitivity, we conduct sensitivity analysis on our modified model. We account for non-linear interactions between parameters by varying all parameters simultaneously using Latin hypercube sampling (*n*=250) over the ranges shown in **Table 2**, and measure sensitivity by calculating Spearman’s rank correlation coefficient (SRCC), ρ, for each parameter against the fold decrease. These simulations use a peptide vaccine dosage of 7 × 10^5^ ng fortnightly, with an iDC dosage of 10^6^ cells/μL injected at the same time as the peptide vaccine. **Table 2** shows SRCC ρ for each parameter.

**Table 2:** Spearman’s rank correlation coefficient between modified model parameters and fold decreases of simulations when varied simultaneously.

Parameter	Description	Range	SRCC
*d_p_*	Peptide decay rate	± 50%	0.0575
*k_p_*	Mature DC uptake rate	± 50%	0.0283
*k_Pi_*	Immature DC uptake rate	± 50%	-0.0825
*k_Pϕ_*	Clearance rate due to splenic macrophages	± 50%	-0.0342
*k_D_*	Mature DC presentation rate	± 50%	-0.0274
*χ*	Concentration of non-vaccine-associated proteins	± 50%	-0.0530
*δ_D_*	Immature DC decay rate	± 50%	0.0198
*d_A_*	Adjuvant washout rate	± 50%	0.1015
*d_D_*	Mature DC decay rate	± 50%	0.0989
*k_S_*	Semi-matured/tolerized DC maturation rate	± 50%	0.0505
*d_m_*	pMHC degradation rate	± 50%	-0.0322
*d_N_*	Naive CTL egress rate	± 50%	-0.1761
*d_NH_*	Naive helper T cell egress rate	± 50%	-0.0490
*d_NR_*	Naive nTreg turnover rate	± 50%	-0.0285
*τ_m_*	DC migration time	± 50%	0.0717
*k_DC_*	Naive CTL-DC interaction rate	± 50%	-0.1214
*V*_tissue_	Volume of tissue site	± 50%	0.0427
*V*_LN_	Volume of lymph node	± 50%	-0.0591
*R*_LH_	Ratio of low-high avidity naive CTLs	10-500	-0.0630
*τ_a_*	T cell division time	± 50%	0.0536
*Φ*_0_	Antigen saturation constant	± 50%	-0.0239
*n_T_*	Number of CTL divisions	10-20	0.4981
*n_H_*	Number of helper T cell divisions	4-10	-0.0036
*n_R_*	Number of nTreg divisions	± 50%	-0.2096
*d_R_*	iTreg decay rate	± 50%	0.0907
*d_RN_*	Effector nTreg decay rate	± 50%	-0.1181
*d_T_*	Effector CTL decay rate	± 50%	0.0497
*μ*	CTL-Treg interaction rate	± 50%	-0.2410
*k_G_*	T cell-growth factor interaction rate	± 50%	-0.0841
*k_R_*	iTreg differentiation rate	± 50%	0.0951
*d_H_*	Effector helper T cell decay rate	± 50%	-0.0119
*r*_1_	Secretion rate of growth signal by CTLs	± 50%	0.0557
*r*_2_	Secretion rate of growth signal by helper T cells	± 50%	0.0508
*d_G_*	Growth factor decay rate	± 50%	0.0774
γ	Growth rate of melanomas	3 × 10^-3^ to 8.7 × 10^−2^/day	-0.7318
*κ*	Carrying capacity of melanomas	48.7 to 2360 k/μL	0.0260
*α*	pMHC regeneration rate	± 50%	0.0925
*k_T_*	Tumor-CTL interaction rate	± 50%	0.0998
*p_T_*	Probability of trogocytosis	± 50%	-0.1336
*ω*_1_	Lysis likelihood for lowest avidity (*j* = 1) CTL	± 50%	0.0588

In our previous model, a sensitivity analysis identified antigen presentation by DCs as a key variable for the beneficial therapeutic value of vaccines. Here, we amend our model with the induction of immature DCs, resulting in supraphysiological levels of DCs. The resulting scale difference reduces the power of DC-associated parameters. Additionally, the model is now sensitive to the tumor growth rate, γ, suggesting that characteristics such as proliferative and apoptotic cell rates may affect the clinical response to the therapeutic vaccine.

## Results

### Modified Mathematical Model

We previously found that the rate of antigen presentation by DCs determined the therapeutic value of an anti-tumor CTL response (16). Here, we hypothesize that inducing high levels of immature DCs would preferentially stimulate naive high-avidity CTLs by increasing the total concentration of mature DCs while lowering the average antigen density per DC. To test this proposed approach, we change Equation 2 in our original model (see Materials and Methods) to include a source term, *v*(*t*), which describes the elicitation of immature DCs, either by injection of ex vivo derived DCs or by recruitment of DC progenitors from the bone marrow *via* cytokine stimulation:

(23) $$\frac{dI}{dt}=s_{D}+v(t)-\delta_{D}I-k_{D}\frac{P}{\chi +P}I.$$

As a simplifying assumption, we assume that induced immature DCs (iDCs) are given at a fixed dose *v*_0_, and at dosing intervals of *ξ* hours *after* the injection of the peptide vaccine, which leads to the functional form:

(24) $$v(t)=v_{0}\sum_{a=0}^{\infty }\delta (t-\xi a).$$

**Figure 1** uses a block diagram to depict the key interactions of our model.

### Increased immature DC levels yields lower peptide:MHC levels and tumor cell reduction

In our example, we assume our tumor is a melanoma and assume that our vaccine either targets either neo-antigen peptides or classical antigens such as MART1. Initially, we simulate the DC context of the vaccine while leaving the peptide dosage fixed at the previously optimized value of 100 ng daily. Using this low peptide dosing, we effectively fix the pMHC levels on DCs to be low. To assess the robustness of our modified model, we next simulated iDC doses ranging from 10^3^ cells/μL to 10^12^ cells/μL, with dosing intervals that range from 0 to 24 hours *after* a peptide injection. For these simulations, we assume that our vaccine adjuvant is delivered at a dose of 10^5^ ng simultaneously with the peptide vaccine.

**Figure 2:**
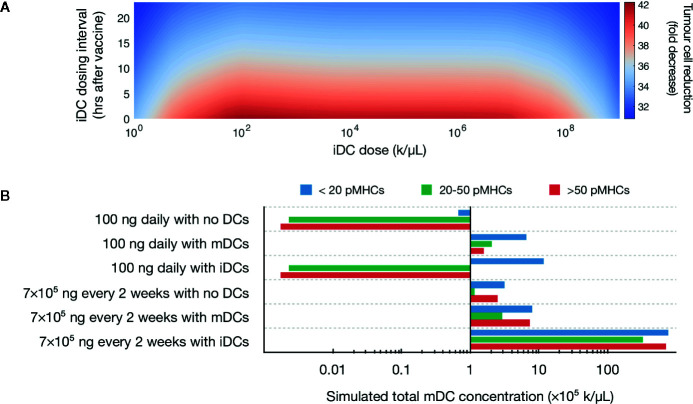
Simulated induction of iDCs favors tumor reduction. **(A)** Heatmap depicts predicted tumor cell reduction (fold change) for different iDC dosages when given with 100 ng of peptide daily. Here, the unit ‘k’ denotes 10^3^ cells. **(B)** Stacked bar chart visualizing the predicted distribution of antigen on mature DCs for various vaccine protocols.

A global sweep of iDC dosages within these ranges identified multiple iDC induction magnitudes as being optimal, i.e., inducing a >90% decrease in tumor burden (**Figure 2A**). For example, an iDC induction magnitude of 10^6^ iDCs/μL given at the same time as the peptide vaccine, induced a 97% decrease in tumor burden. Importantly, the substantial reduction in tumor concentration we observed is neither dose dependent nor time dependent within our parameters, with a wide range of iDC concentrations and dosing intervals achieving a high degree of tumor reduction. Indeed, for iDC doses between 10^2^ to 10^7^ k/μL, the *percentage decrease* in tumor concentration varies minimally from the local optimum regardless of the dosing interval used. We thus find that the temporal robustness of this system centered around iDC induction and high-avidity T cell induction potentially allows for the possibility of introducing other combinatorial therapeutic strategies that may synergize with vaccine strategies, including checkpoint blockade and inducers of immunogenic cell death.

Our initial results demonstrated that increased iDC levels, rather than increased mDC levels, favor robust tumor clearing. We next set to determine if similar results could be recapitulated with clinically relevant vaccine dosages, rather than the 100 ng daily peptide dose identified by our previous model. We first compared pMHC levels in three therapeutic variations: peptide with either no DCs, induction of iDCs, or induction of mDCs with around 6 × 10^6^ DCs (which, for a control volume of *V*_LN_ =4.2 μL, equates to a concentration of 1.43 × 10^3^ k/μL), a dosing concentration similar to previously used in a clinical setting and within optimal concentrations found in our global sweep above (80). We assume that within this population of ex vivo matured DCs (mDCs), pMHCs are normally distributed with mean *μ*=100 and variance *σ* ^2^ = 25 (76). Additionally, we compare peptide dosing concentrations for both an ideal 100 ng daily and a clinically relevant 7 × 10^5^ ng every 2 weeks (20). Our model shows that at both peptide doses, induction of iDCs results in increased pMHC-low mature DCs as compared to no DC or mDC conditions (**Figure 2B**). This reduced antigen density in the context of the same peptide injection concentrations is due to the significantly increased numbers of mDCs generated by inducing iDCs (**Figure 2B**). These increased numbers are due to the longer half-life of iDCs as compared to mDCs, which are thought to rapidly decay upon maturation. As a result, the same peptide concentration dispensed over a larger number of DCs results in lower pMHC levels per DC.

### Immature DCs Promote High-Avidity T Cells and Tumor Clearance in Clinically Relevant Dosing Schemes

**Figure 3:**
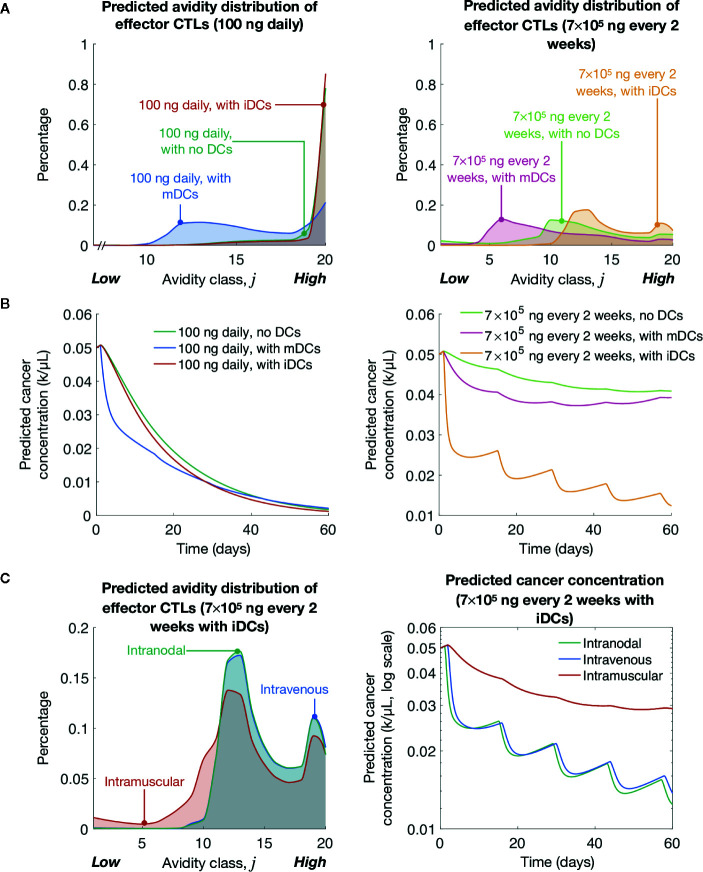
Simulated induction of iDCs at clinically relevant vaccination doses yields significant tumor cell clearance. **(A)** Simulated avidity distribution of effector T cells for various vaccine protocols. **(B)** Simulated cancer concentrations over time for various vaccine protocols. **(C)** Simulated avidity distribution of effector T cells and tumor cell reduction (fold change) for different delivery routes. The unit ‘k’ denotes 10^3^ cells.

Previously, we showed lower levels of pMHC competitively favor the expansion of high-avidity T cells rather than low-avidity T cells (16). As expected, we find that at both peptide dosing schemes induction of iDCs significantly favors the generation of high-avidity T cells compared to mDCs (**Figure 3A**). The optimal low dose of 100 ng daily of peptide significantly favors the development of high-avidity T cells, but even with the clinically relevant dosing of 7 × 10^5^ ng every 2 weeks, the induction of iDCs significantly shifts the balance of T cell composition to favor high-avidity T cells. This highlights that while traditional mDC or peptide-only vaccination strategies do increase T-cell induction, they do so at the expense of high-avidity T cells. In reflection of increased expansion of high-avidity T cells, our simulations further demonstrate that iDC induction results in improved cancer cell lysis (**Figure 3B**).

Finally, we compared vaccine responses in three different delivery routes: intramuscular, intravenous, and intranodal. For intramuscular case, at most, only 4% of DCs are expected to arrive at the LN (81). To model this, we multiply the source terms (described by Eqs 17–20) by 4%. To model intravenous delivery, we modify our initial conditions so that DCs in our model are characterized by blood DC data (75). Finally, to model intranodal delivery, we assume there is no migratory delay between the injection site and the LN (i.e., τ*_m_* = 0), and that there is no volume change between DC compartment and the LN compartment. Our simulations suggest that in the context of avidity-selection and tumor clearance (see **Figure 3C**), DC vaccination route is a critical consideration for maximizing vaccine efficiency. iDCs intranodal injections followed by intravenous injection were both preferable over intradermal DC injections. This data highlights the importance of high iDC cell numbers accumulating in the LN in our vaccination model.

## Discussion

Cancer immunotherapy is now a routine means of successfully treating tumors of various types in the clinic. However, improved immunotherapies to benefit greater numbers of patients with increased durability are still needed. Despite its tremendous successes, ICB therapy only benefits less than the majority of patients treated (82–84) and presents significant risks for adverse side-effects (85–87). Therapeutic peptide vaccines can robustly induce a tumor-specific CTL response with limited side effects due to induction of an antigen-specific immune response rather than broad immune activation (20). Preferential development of high avidity anti-tumor CTLs enables enhanced tumor cell killing (9, 18). Previously, we showed that vaccine dosages could be optimized to preferentially elicit high-avidity CTLs, unlike standard dosages that elicit low-avidity CTLs (16). In that study, we showed that the efficacy of a dosage-optimized approach depended on DC-related parameters, which motivated us to explore how we could harness immature DCs to boost anti-tumor activity.

High peptide antigen doses have been shown experimentally to result in low avidity and T cell responses (88, 89). However dosing timing strategy has been shown to have a significant effect on the average avidity of a T cell population (90, 91). Other work has shown that modulation of antigen presenting cells is a key component of the induction of high avidity T cells (92). We hypothesized that increasing the magnitude of iDCs given with a dosage-optimized peptide vaccine may enhance CTL responses. It is important to stress that this approach is conceptually different from traditional DC vaccines in which ex vivo matured DCs are injected (3, 17). To assess this approach, we extended our previous model to account for a hypothetical induction of iDCs. We show that induction of iDCs, and not mDCs, can significantly reduce tumor burden, improving upon the performance of a peptide vaccine. A key assumption of our model is that iDCs will have a longer half-life and inducing iDCs will result in a larger overall pool of DCs as compared to the injection of mDCs, which are known to have a shorter half-life (62). Our simulations show that these effects are tied to the increased half-life of iDCs and therefore increased DC levels in general, which results in a lower average antigen density per DC. As such, induction of iDCs favors the preferential stimulation of high-avidity CTLs and tumor cell clearance. In support of our findings, increased circulating DC levels have been associated with increased survival in certain malignancies (93–95). Further experimental or clinical evidence of the relationship between circulating DC levels and vaccination efficacy is needed.

Early cancer vaccines targeting over-expressed CAAs such as MART-1, MAGE, NYE-ESO-1, HER2, and MUC-1 demonstrated mediocre clinical results. Evidence suggests that the T cell repertoire capable of responding to these antigens are primarily composed of low-avidity T cells due to central tolerance of T cells specific for self-antigens (96). Recently, there has been renewed interest in cancer vaccines due to promising results for those targeting neoantigens (97–100). Additionally, encouraging preliminary clinical results have recently been observed in therapeutic approaches combining DC vaccines with checkpoint blockade (101). Our findings suggest that inducing increased iDC levels would benefit vaccines targeting either over-expressed CAAs or neoantigens, as the expansion of high-avidity CTLs would favor clinical responses in both scenarios.

Initial DC vaccines, such as Sipuleucel-T, were major milestones for immunotherapy-based treatments of cancer and demonstrated modest, but meaningful, clinical results (102). While DC vaccines have not achieved widespread therapeutic success, it is unclear if this is a result of targeting TAAs, the influence of previously unknown immunosuppression mechanisms in the tumor microenvironment, or difficult in manufacturing cell products (103). While traditional DC vaccines have been based on ex vivo antigen loading and maturation of autologous DCs, our model finds that injecting iDCs results in a maximal anti-tumor response. We find that intranodal injection, as compared to intradermal or intravenous injection, results in the most T cell activation as it ensures high numbers of DCs are loaded with low pMHC levels. Although repeated intranodal injection of iDCs is not an ideal clinical scenario, it highlights the importance of recent bioengineering efforts to localize tumor antigen vaccination to lymph node sites (104). However, intravenous injection of iDCs did result in substantial tumor burden reduction. We suggest that other alternatives to iDC generation and injection, such as mobilization of bone marrow DC precursors, is an attractive possibility for future consideration in tumor vaccine design. Treatments with cytokines such as Fms-like tyrosine kinase 3 ligand (Flt3L) has demonstrated efficacy in increasing levels of circulating DCs (105–107). Our model suggests that elevated levels of iDCs, rather than mDCs, favors a longer half-life of the circulating DC compartment and results in lower average pMHC levels that would then favor high-avidity T cell generation. Therefore, induction of iDCs by any of several means followed by peptide vaccination and adjuvant for *in vivo* DC maturation would favor tumor clearance. While our model simplistically accounts for adjuvant as a necessary requirement for DC maturation and activation of T cells, we acknowledge that different adjuvant choices may have highly variable effects on DC activation and downstream T cell differentiation (108, 109).

Our work addresses an important and less appreciated element of cancer vaccines – how vaccine design and administration can select for and enhance the proliferation of high-avidity CTLs. However, there remain many barriers to efficacy with a combination strategy that our model does not consider. For example, we do not account for potential intra-tumoral heterogeneity of antigen expression, factors influencing CTL trafficking to tumor sites, or a multitude of potential immune suppression mechanisms found within tumor microenvironments. Additionally, in modelling the T cell activation we do not *explicitly* model TCR signaling. Future work will involve incorporating existing validated models of TCR signaling (110), and calibrating these models to avidity data from (111, 112). Defining the minimum complexity of the immune system is challenging, and the model used in this study does not, nor does it aim to account for all known immune interactions.

The mathematical model presented here proposes that increasing the magnitude of iDCs with an optimized peptide vaccine may improve tumor clearance. The model highlights the relative importance of antigen loads on DCs, which facilitate the selective expansion of high-avidity CTLs. While pre-clinical experimental validation of our findings are necessary, our model suggests previously unappreciated aspects of vaccine design that may be necessary for the development of effective cancer treatments.

## Data Availability Statement

All datasets presented in this study are included in the article.

## Author Contributions

AK performed the simulations and formal analysis. AK and CE wrote the first draft of the manuscript. All authors contributed to the article and approved the submitted version.

## Funding

This work was supported by an Australian Government Research Training Program Scholarship; an Australian Research Council Discovery Project [DP180101512]; and by the US Department of Defense Breast Cancer Research Program [W81XWH-11–1–0548].

## Conflict of Interest

The authors declare that the research was conducted in the absence of any commercial or financial relationships that could be construed as a potential conflict of interest.
